# Naturally Occurring Oxazole-Containing Peptides

**DOI:** 10.3390/md18040203

**Published:** 2020-04-10

**Authors:** Jessica T. Mhlongo, Edikarlos Brasil, Beatriz G. de la Torre, Fernando Albericio

**Affiliations:** 1 Peptide Science Laboratory, School of Chemistry and Physics, University of KwaZulu-Natal, Durban 4001, South Africa; jtmhlongo91@gmail.com (J.T.M.); MacedoBrasilE@ukzn.ac.za (E.B.); 2 KwaZulu-Natal Research Innovation and Sequencing Platform (KRISP), School of Laboratory Medicine and Medical Sciences, College of Health Sciences, University of KwaZulu-Natal, Durban 4041, South Africa; 3 CIBER-BBN (Networking Centre on Bioengineering, Biomaterials and Nanomedicine) and Department of Organic Chemistry, University of Barcelona, 08028 Barcelona, Spain

**Keywords:** marine natural products, solid-phase peptide synthesis, heterocycles

## Abstract

Oxazole-containing peptides are mostly of marine origin and they form an intriguing family with a broad range of biological activities. Here we classify these peptides on the basis of their chemical structure and discuss a number of representatives of each class that reflect the extraordinary potential of this family as a source of new drugs.

## 1. Introduction

Peptides play crucial and diverse biological roles as signaling and regulatory molecules in various physiological and pathological processes, such as defense, immunity, stress, growth, homeostasis, reproduction, and other cell functions [1]. Due to their optimized synthesis, bio-specificity, and efficacy profile in humans, peptides provide a platform for the design of novel therapeutic drugs [2,3]. However, the use of these molecules as therapeutics is limited because of their cell membrane and blood–brain barrier impermeability, poor chemical and physical stability, and short plasma half-life [4]. 

These limitations have brought about a systematic search for peptidomimetics [5,6], such as oxazole-based peptides. Oxazoles are an important class of 5-membered *N,O*-heterocyclic compounds with numerous applications, from medicines to agrochemical products. The presence of oxazole moieties in natural peptides confers stability and/or electronic distribution to the peptide chain, thereby enabling peptide–protein recognition and DNA/RNA–peptide interactions [7]. Furthermore, oxazoles show antibacterial [8,9], antiviral [10], antimalarial [11], and anti-algal properties [12], as well as cytotoxic activity [13], among others [14]. 

**Scheme 1:**
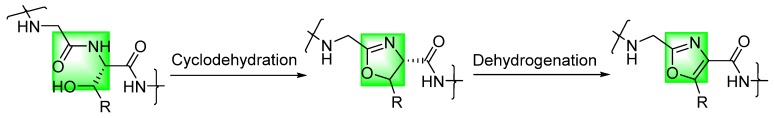
Biosynthesis of oxazoles.

Oxazole-containing peptides are mostly of marine origin and can be formed via ribosomal and non-ribosomal mechanisms, as well as through synthetic means. The biosynthetic pathways, either via ribosomal or non-ribosomal processes, involve the heterocyclization of β-hydroxyl residues, mainly serine (Ser)/threonine (Thr), into an oxazoline moiety, followed by an oxidation step catalyzed by flavin mononucleotide (FMN)-dependent dehydrogenase (Scheme 1). In addition, several synthetic methodologies to prepare oxazole rings have been reported in detail [15,16,17].

Here we review the isolation, structural elucidation, and biological activity of some representatives of each class of the natural oxazole-containing peptides: short linear, long linear, cyclic, bicyclic, and thio peptides. 

The division between small and long peptides (Section 3) has been decided by the authors and applies only to this work.

## 2. Short Linear Peptides 

### 2.1. Almazoles A–D

**Figure 1:**
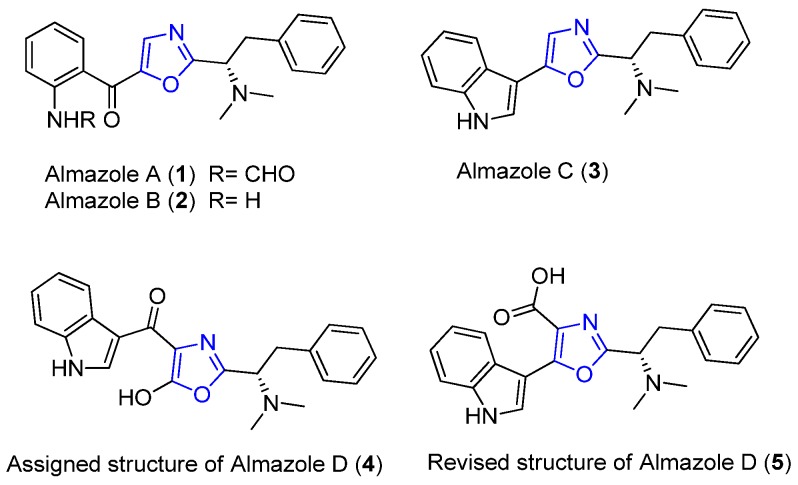
Structures of Almazoles A–D.

Between 1994 and 1996, N’Diaye et al. [18,19] and Guella et al. [20] reported the dipeptide 2,5-disubstituted oxazoles A–D (**1**–**4** in Figure 1), unusual structures isolated from Delesseriaceae marine alga, found on the coast of Senegal. Named almazoles by the authors, the structures of **3** and **5** show an indole moiety. The stereochemical assignment was determined by biomimetic synthesis. However, for the original assigned structure of **4**, a revised structure of this compound (**5**) via chemical synthesis was reported [21]. Compound D (**5**) differs from **3** in that its biogenesis conceivably involves oxidative deamination rather than decarboxylation of Trp, hence rationalizing both the extra carbonyl group and the hydroxyl group at the oxazole ring. No biological testing has been reported for **1**–**3**. Regarding **5**, it showed potent antibacterial activity against Gram-negative *Serratia marcescens* and *Salmonella typhi* [19].

Lade et al. also synthesized the two stereoisomers of **5** and evaluated their anti-tuberculosis activity. The hybrid 5-(3-indolyl)oxazole scaffold of these compounds displayed drug-like properties and revealed promising activity against *Mycobacterium tuberculosis* (Almazole D (**5**), MIC = 100 μM; R-enantiomer, MIC = 12.5 μM) [22].

### 2.2. Martefragin A

**Figure 2:**
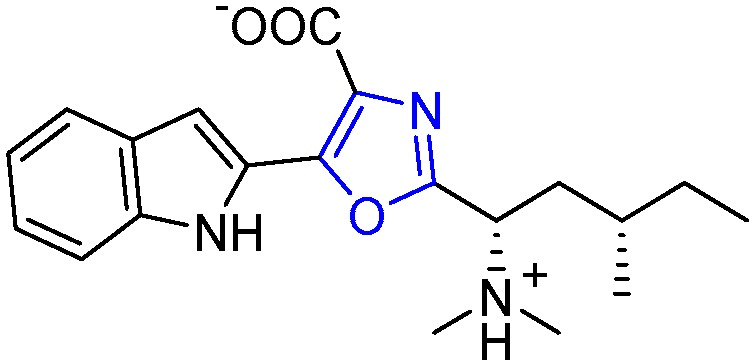
Structure of Martefragin A.

Takahashi et al. isolated the secondary metabolite named Martefragin A (**6**) from the red algae *Martensia fragilis* Harvey (Figure 2) [23]. The structure features a trisubstituted 2,4,5-oxazole ring, in which the indole moiety, originated from a β-hydroxyl-Trp group, is directly linked to C5, a carboxylate group at C4 and a branched five-carbon side chain bearing two stereocenters at C2. The structural assignment was established based on spectroscopic and X-ray crystal analyses, and the absolute configuration of the stereocenter carbon atoms was determined through total synthesis studies [24]. Compound **6** has been reported to be an inhibitor of lipid peroxidation in biological systems [23].

### 2.3. Muscoride A

**Figure 3:**
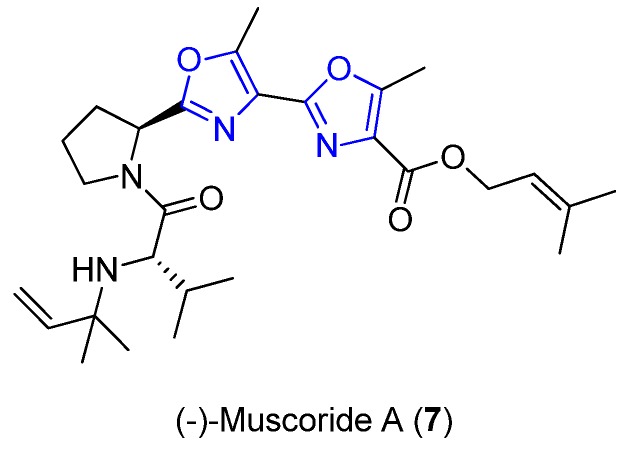
Structure of (-)-Muscoride A.

(-)-Muscoride A (**7**) is an oxazole peptide alkaloid that was isolated by Nagatsu et al. from terrestrial freshwater cyanobacterium *Nostoc muscorum* (Figure 3) [25]. Featuring a bis-5-methyloxazole moiety and a prenyl-like functionality at the α-nitrogen atom of a Val residue, the structure of **7** has a unique chemical architecture among the class of alkaloids. The construction of the bis-oxazole rings has been proposed to arise from two Thr units that undergo a sequence of cyclodehydration–oxidation reactions [26]. In addition, unlike some reported indole-based natural products that commonly show the presence of the reverse prenyl functional group, this secondary metabolite is among the first bisheterocyclic-oxazole alkaloids to have such functionality (*N*-(2-methyl-3-buten-2-yl). Although **7** has shown only modest antibiotic activity, and no further biological tests have been performed, several research groups have used it as a platform to test the efficiency of a series of synthetic methodologies for the regioselective assembly of oxazole compounds [27]. To this end, the total synthesis of **7** was achieved soon after its first isolation by Nagatsu et al., thereby providing additional structural information about the initial unresolved stereochemistry of the molecule, such as its relative and absolute configuration [28].

## 3. Long Linear Peptides

### 3.1. Microcin B17

**Figure 4:**
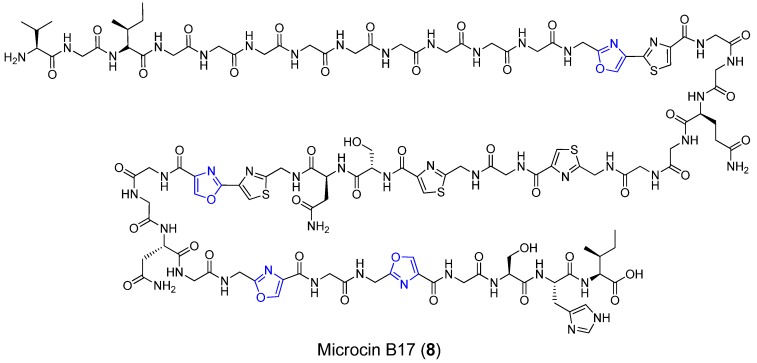
Structure of Microcin B17.

Microcin B17 (MccB17) (**8**) is a post-translationally modified peptide that was isolated from *Escherichia coli* strains (Figure 4) [29]. Of the 43 amino acids comprising its structure, the peptide backbone contains 20 Gly units, and 14 amino acids are post-translationally modified, represented by the eight 5-membered heterocycles—oxazole/thiazole rings. Genetic studies of MccB17-producing strains revealed that, of the seven operon genes involved in the biosynthesis of this peptide, at least three gene products code for modifications of certain Ser and Cys residues in the heterocyclic backbone residues, including the two 4,2-fused heterocyclic rings [30]. A full structural characterization of **8** was carried out using several analytical techniques, as well as 2D NMR spectroscopy and ^15^N-labeled peptides [31]. This natural Gly-rich polypeptide belongs to a class of DNA-gyrase inhibitors. Other classes of these inhibitors include quinolones and coumarins [30,31]. Evaluation of the bioactivity of **8** against *E. coli* cells suggested potent antibacterial activity by targeting the double-strand DNA, ultimately leading to cell death. However, a series of mutagenesis experiments indicated that alteration of the number of rings, their chemical nature, and their position along the peptide chain directly affect the antibiotic efficacy of this compound [7]. In this regard, substitutions of the 4,2-tandem bisheterocycle (thiazole-oxazole) moiety with 4,2-fused bisheterocycles ((oxa)thiazole-(oxa)thiazole) in the native MccB17 structure had a significant effect on the biological activity of the compound. The possibility of designing a library of MccB17 analogs for further biological evaluation led synthetic chemists to work on the total synthesis of this natural product, which has been accomplished successfully [31,32,33]. 

### 3.2. Plantazolicins A and B

**Figure 5:**
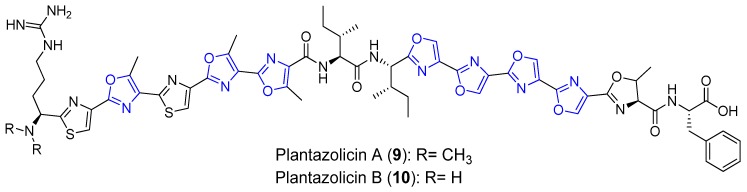
Structures of Plantazolicin A and B.

Plantazolicins A (**9**) and B (**10**) (Figure 5) isolated from *Bacillus amyloliquifaciens* FZB42 have biosynthetic pathways resembling that of Microcin B17 (**8**), and they are classified as thiazole/oxazole-modified microcins (TOMMs). The biosynthetic gene cluster machinery of Plantazolicin consists of 12 genes that are involved in the synthetic process of this secondary metabolite, as well as in modification, export, and self-immunity mechanisms. Specifically, the heterocyclization of Cys and Ser/Thr residues into the azole rings highlights a series of post-translational modifications, which are encoded by a trimeric BCD protein complex (a cyclodehydratase (C), a dehydrogenase (B), and a docking protein (D)) [34].

Structures **9** and **10** have a limited number of protons, due to the presence of ten heterocycles within their structures plus a phenyl group. To determine the molecular structure of these compounds, ^15^N-labeling experiments were undertaken, together with a series of other spectroscopic techniques. On this occasion, two important questions were raised during structural assignment: (i) whether the enzymatic dimethylation step of **9** and **10** installed the methyl groups at the N-terminal amine or at the alkyl side chain of an Arg residue, and (ii) the position of the exact site of the oxazoline heterocycle. These questions were addressed using the mass spectrometry technique known as collision-induced dissociation (CID). It was observed that the N-terminal ion fragment contained the two post-translational methyl groups, and the C-terminal fragment showed the oxazoline moiety [35,36]. When screened on a variety of Gram-positive/Gram-negative organisms, **9** displayed discriminating antibiotic activity against *Bacillus anthracis*, but no activity on Gram-negative organisms [34,35].

### 3.3. Goadsporin 

**Figure 6:**
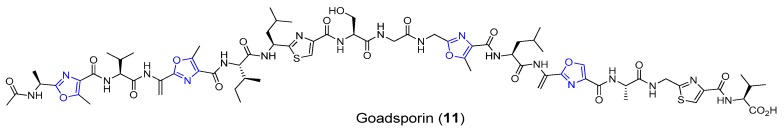
Structure of Goadsporin.

In 2001, Onaka et al. reported the isolation and characterization of Goadsporin (**11**) from *Streptomyces* sp. TP-A0584 (Figure 6). According to NMR analysis, the structure of **11** consists of 19 amino acids (ATVSTILCSGGTLSSAGCV), with AT/ST/GT/SS pairs of L-amino acids, resulting in the formation of four oxazole rings and the condensation of LC/GC amino acids, thereby furnishing two thiazole units. The structure also shows an acetylated *N*-terminal and two dehydroalanine residues, which are formed by glutamylation of Ser, followed by glutamate elimination, as suggested by a recent study [37]. Finally, the L-configuration of the amino acids was determined by Marfey’s method. Compound **11** belongs to the class of ribosomally synthesized and post-translationally modified peptides (RiPPs) and it has potent biological properties against *Streptomyces* spp. and *S. scabies*, which is the organism that causes potato scab. When screened on a range of other *Streptomyces* strains, **11** induced the formation of several other secondary metabolites and also caused sporulation. These properties have generated interest in the total synthesis of **11**, which has been successfully accomplished [38]. 

## 4. Cyclic Peptides

### 4.1. Bistratamides C, D, G, H, I, M, and N

**Figure 7:**
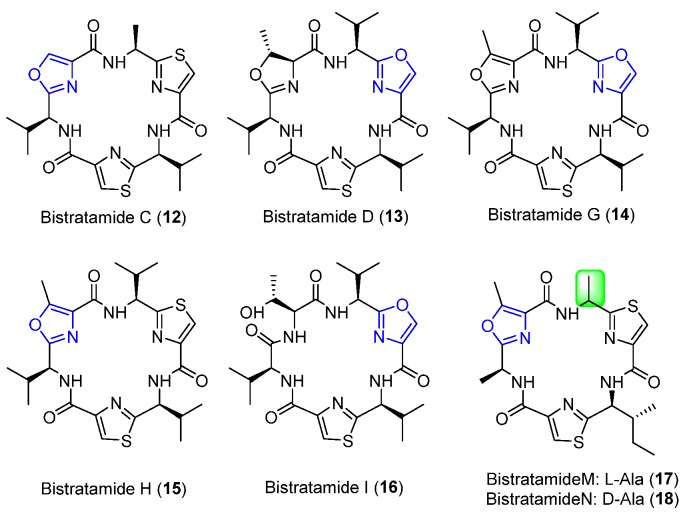
Structures of Bistratamide C, D, G, H, I, M, and N.

Bistratamides C, D, G, H, I, M, and N (**12**–**18**) are cyclic hexapeptides that were isolated from the cytotoxic extract from the ascidian *Lissoclinum bistratum* (Figure 7) [39,40]. Sharing a similar cyclic backbone, the structures of **12**–**18** were determined by means of 2D NMR spectroscopy, as well as mass spectrometry. The metabolites **12** and **13** from the northern Philippine ascidian *L. bistratum,* studied by Ireland’s group in 1992, show one oxazole/two thiazole rings, and one oxazoline/one thiazole/one oxazole ring in their structures, respectively. Regarding the amino acid residues present in **12** and **13**, the former has one Ala and two Val residues and the latter three Val residues. When introduced directly into the central nervous system (CNS) of mice, **13** exhibited neurodepressant properties [41]. 

A decade later, Bistratamide G–I (**14**–**16**) were reported in the literature [39]. In addition to being isolated from the same organism as **12** and **13**, compounds **14**, **15**, and **16** all have three Val residues. However, they differ from one another in the number and type of residues that give rise to the heterocycles oxazole and thiazole, and **16** is the only compound with a Thr unit. Bistratamides **14**, **15**, and **16** were found to be moderately cytotoxic in an HCT-116 cell line assay [39]. 

Two additional bistratamides with antitumor activity, M (**17**) and N (**18**), were also characterized, as reported by Urda et al. Overall, the ^1^H NMR data of **17** and **18** presented a similar pattern to that of Dolastatin E (**30**) [40]. Of note, the structures of the peptides westiellamide [42], Dendroamides **19**–**21** [43], and Nostocyclamides **22** and **23** [12] are all related to Bistratamides **12**–**18**. 

### 4.2. Dendroamides A–C 

**Figure 8:**
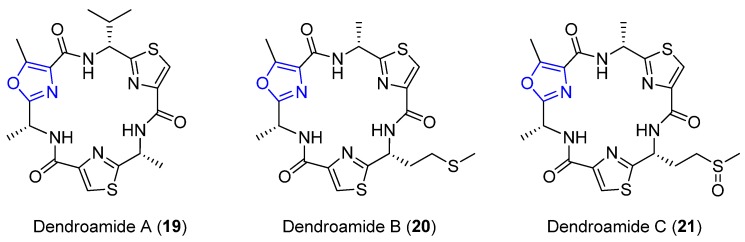
Structures of Dendroamide A–C.

First reported by Ogino et al., Dendroamides A–C (**19**–**21**) are bistratamide-type cyclic hexapeptides that were isolated from the terrestrial blue-green alga *Stigonema dendroideum* Fremy (Figure 8). The structures of **19**–**21** were fully characterized as featuring one methyloxazole, two thiazole moieties, and three amide groups, from which an 18-membered cycle is formed. Although having the same basic skeleton—as determined by chemical degradation, NMR, and EIMS experiments—**19**–**20** differ from one another in the type of amino acid side-chain residues. In this regard, Val, Met, and methionine sulfoxide (Met(O)) groups are present in Dendroamides A, B, and C, respectively. Dendroamide A (**19**) was evaluated as a potent MDR-reversing agent against breast carcinoma (MCF-7/ADR) cells, while **20** and **21** showed no activity. The total synthesis of **19** has been successfully accomplished [44,45,46]. 

### 4.3. Nostocyclamides

**Figure 9:**
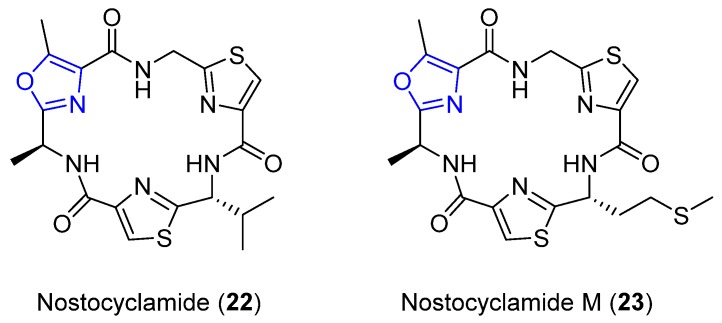
Structures of Nostocyclamides.

Nostocyclamide (**22**) and Nostocyclamide M (**23**) (Figure 9) were isolated by Todorova et al. from the freshwater cyanobacterium *Nostoc* 31. The former has oxazole/thiazole rings linked by Gly, Val and Ala residues, while in the latter they are linked by Gly, Ala, and Met. Structural assignment based on 2D NMR data and ^15^N labeling experiments revealed three heterocycles (an oxazole ring and two thiazole rings), including the other substituents highlighted above. The absolute configuration of the methine-Ala and methine-Val carbons, as *S* and *R*, respectively, was determined through X-ray crystallographic studies. Both **22** and **23** have cyanobacterial and allelopathic activity, and the former also shows anti-algal activity [12,47].

### 4.4. Tenuecyclamides A–D

**Figure 10:**
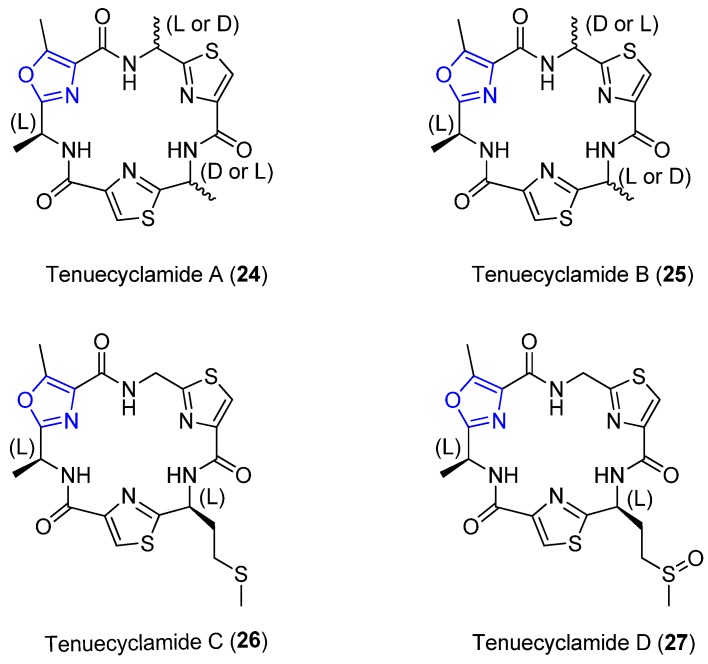
Structures of Tenuecyclamide A–D.

Tenuecyclamides A–D (**24**–**27)** (Figure 10) were isolated as patellamide-like compounds from the *Nostoc spongiaeforme* var. *tenue* Rao (Nostocales, Nostocaceae), collected in Bet Dagan, Israel, by Carmeli et al. The planar structures of **24**–**27** were determined by 2D-NMR techniques and high-resolution mass spectrometry measurements. The MeOH extract of the freeze-dried cyanobacterium showed antimicrobial activity (against *Bacillus subtilis* and *Staphylococcus aureus*) and inhibited the division of embryos of the Mediterranean sea urchin. Compounds **24** and **25** contain three Ala residues, two thiazoles and one methyloxazole; however, they differ from each other in the stereochemistry of the Ala residues. Compound **26**, in turn, contains Gly, Ala, and Met, two thiazoles and one methyloxazole. Marfey’s method failed to fully solve the stereochemistry of **24** and **25**. However, for **26** and **27**, the stereo-assignment confirmed the L-forms of the amino acid residues [48]. 

### 4.5. Venturamides A and B 

**Figure 11:**
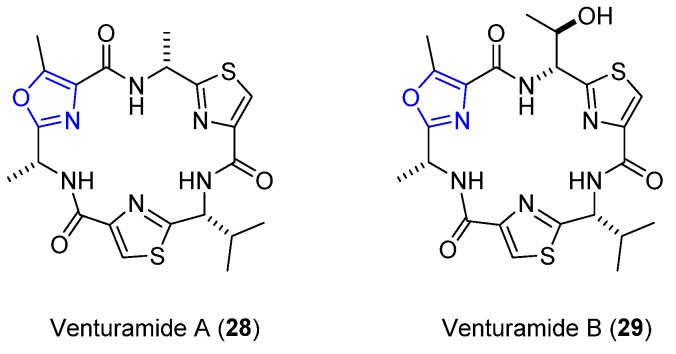
Structures of Venturamide A and B.

Using antimalarial bioassay-guided fractionation, Linington et al. isolated Venturamide A (**28**) and B (**29**) from the marine cyanobacterium *Oscillatoria* sp. (Figure 11), collected in Buenaventura Bay (Colombia). Both **28** and **29** showed strong in vitro activity against *Plasmodium falciparum* and mild cytotoxicity to mammalian Vero cells. They have also shown mild activity against *Trypanasoma cruzi*, *Leishmania donovani*, and MCF-7 cancer cells. The structures were characterized by 1D and 2D NMR spectra, which revealed that **28** has a Val residue and two thiazole rings. On the other hand, the structural characterization of **29** unveiled that it contains Thr in the place of Ala, adjacent to the thiazole ring. Marfey’s analysis showed that all the amino acids of these two peptides have a D-configuration [11].

### 4.6. Dolastatins E and I 

**Figure 12:**
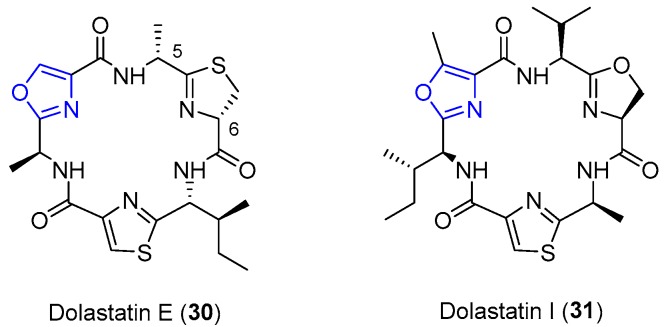
Structures of Dolastatin E and I.

The macrocyclic hexapeptides Dolastatin E (**30**) and I (**31**) were obtained from the sea hare *Dolabella auricularia* by Ojika et al. and Sone et al. (Figure 12), respectively [49,50]. Remarkably, in spite of being isolated from distinct organisms, the natural cyclic peptides Bistratamides **12**–**18**, Raocyclamides **32a** and **32b**, Nostocyclamides **22** and **23**, and Dendroamides **19**–**21** are among the 18-membered macrocycles with structures similar to those of Dolastatins **30** and **31**. One major difference between the chemical structure of Dolastatin **30** and the structures in the group of the hexapeptides outlined above, including Dolastatin **31**, is the presence of a semi-oxidized heterocyclic thiazoline, post-translationally modified from Cys and Ala residues [49]. To fully characterize the stereochemistry of **30**, which could only be partially assigned as 5*R*/6*S* by acid-catalyzed hydrolysis and ozonolysis experiments, the total synthesis of this metabolite was necessary. In this regard, Nakamura et al. synthesized **30** and observed that it contained the following amino acid units with their respective stereochemical assignment: L-Ala, D-Ala, *allo*-D-Ile, and D-Cys. However, no chirality was determined for Ser or Cys, as these residues underwent heterocyclization to form the oxazole and thiazole rings, respectively [51].

Conventional ^1^H-^1^H/^1^H-^13^C NMR correlations and mass spectral data revealed that the structure of **31** contains two heteroaromatic rings and one oxazoline moiety, as well as Val, Ile, and Ala residues. By subjecting this compound to chemical degradation conditions, it was observed that all the amino acids in the structure adopted the L-configuration. Compounds **30** and **31** showed weak cytotoxicity on tumor HeLa-S3 cells [50]. 

### 4.7. Raocyclamides A–B 

**Figure 13:**
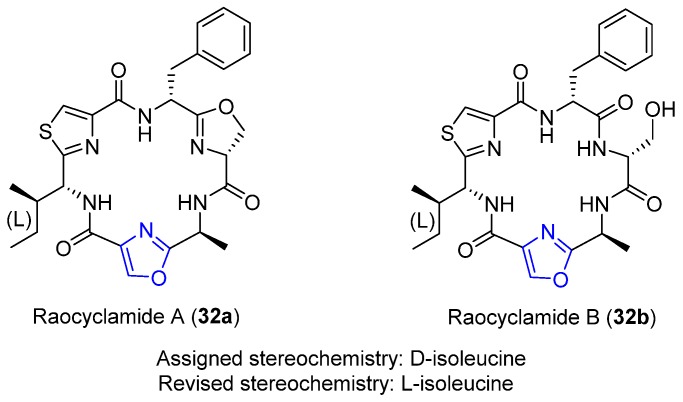
Structures of Raocyclamide A and B.

In the search for versatile structures from cyanobacterium *Oscillatoria raoi* with potent biological activities, Admi et al. isolated Raocyclamides A (**32a**) and B (**32b**) (Figure 13). The structures of these two cyclic hexapeptides share the same number of amino acid residues (Ala, Ser, Phe, Cys, and Ile), as indicated by chemical degradation experiments, as well as spectroscopic and MS data. The structure of **31** differs from that of **32** in that it has three heterocyclic units, among which a 5-membered *N,O*-based heterocycle appears in its oxazoline oxidation state. In **32**, the oxazole and the thiazole rings are post-translationally modified but no modification is observed between Ser and the carbonyl group of Phe. Compound **31** is moderately cytotoxic against sea urchin embryos. No further bioactivity has been reported for **31** or **32** [52]. The total synthesis of **31** and **32** was undertaken by Freeman et al., and it was concluded that their structures contained L-Ile rather than D-isoleucine, as previously reported [53]. 

### 4.8. Microcyclamides 

**Figure 14:**
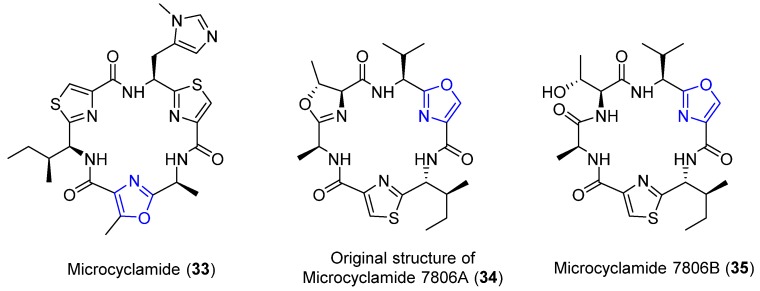
Structures of Microcyclamides.

In 2000, Ishida et al. isolated the first cyclic hexapeptide microcyclamide (**33**) from the freshwater cyanobacterium *Microcystis aeruginosa* NIES-298 (Figure 14). The structural assignment was determined by 2D NMR spectroscopy, revealing the following fragments: a thiazole-methyl histidinyl; a thiazole-isoleucinyl; and a methyloxazole-alanyl. Additionally, ozonolysis/hydrolysis experiments and Marfey’s analysis fully established the stereochemistry of the structure of **33**. Biological evaluation of **33** revealed cytotoxicity towards P388 murine leukemia cells at IC_50_ 1.2 μg/mL [54]. 

Other cyclic hexapeptides were subsequently brought to light, such as Microcyclamide 7806A (**34**) and 7806B (**35**) (Figure 14), reported by Ziemert et al. in 2008, upon comparing the biosynthetic gene clusters of the organisms *M. aeruginosa* NIES-298 and *M. aeruginosa* PCC-7806 [55]. 

**Scheme 2:**
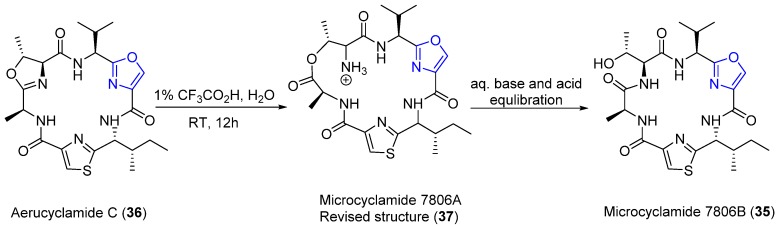
Acidic and basic hydrolysis of Aerucyclamide **36**.

However, in that same year, Portmann et al. published the isolation of Aerucyclamide C (**36**) and the structural revision of Microcyclamide 7806A (**37**). Regarding the revised structure of **37**, the authors observed that while assigning the structure of Aerucyclamide **36** by NMR, most of the chemical shifts obtained were in line with those studied by Ziemert et al. [55] in the structure of Microcyclamide 7806A (**37**), the exception being the appearance of a broad signal resonating at 8.28 ppm in the latter. To better understand this mismatch between the structures of **34** and **36**, Portmann et al. analyzed the resulting product, structure **37**, from the acidic treatment of **36.** They concluded that the signal at 8.28 ppm corresponds to the protons of the NH_3_^+^ group, which results from the oxazoline-ring opening upon acidic hydrolysis. Further evidence to support the existence of the NH_3_^+^ group was provided by HR-ESIMS data. Finally, the NMR data of **37** were in accordance with those of Microcyclamide 7806A (previously assigned structure **34)**. This outcome led the authors not only to reassign the original structure of **34** to the revised structure **37,** but also to analyze Microcyclamide 7806B (**35**), furnished from **37** under a sequence of basic and acidic conditions (Scheme 2). Therefore, the structures of **35** and **37** are in fact hydrolysis products of **36**, as opposed to being natural products from *M. aeruginosa* PCC 7806. The observation that **37** and **35** lacked bioactivity in a range of bioassays, including antibacterial activity and others, further demonstrates their synthetic nature, i.e., **35** and **37** are unnatural cyclamides, in contrast to the natural cyclamides that normally show a wide spectrum of biological activity. In this regard, **36** displayed relevant activity against *P. falciparum* K1, with an IC_50_ value of 2.3 μM, and emerges as a promising antimalarial agent [56]. The activity of **36** vs. the lack of activity of **35** and **37** reinforces the importance of the heterocycle (oxazoline in this case) for the activity due presumable to the conformational restriction imposed by the cycle.

### 4.9. Leucamide A 

**Figure 15:**
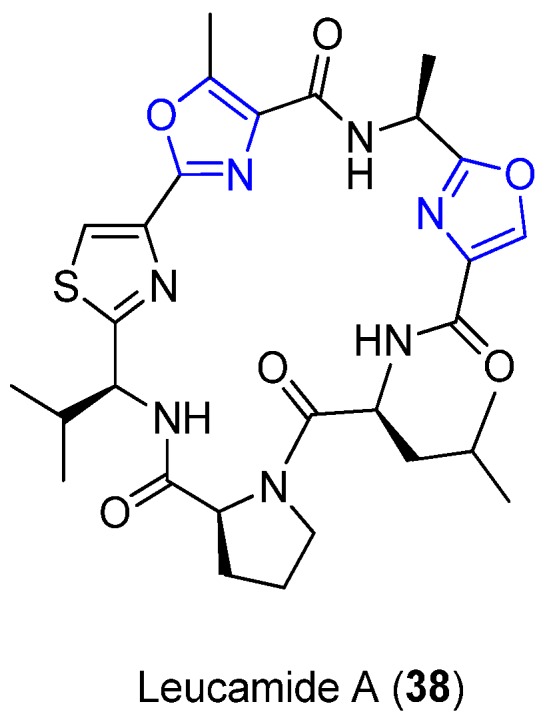
Structure of Leucamide A.

Leucamide A (**38**) was first isolated by Kehraus et al. from the marine sponge *Leucetta microraphis*, collected from the Great Barrier Reef, Australia (Figure 15). The planar structure of **38** was analyzed by spectroscopic techniques, revealing seven amino acids and other amino acid derivatives: Leu, oxazole, Ala, methyloxazole, thiazole, Val, and Pro. Interestingly, the rare mixed 4,2-bisheterocycle tandem pair consists of a methyloxazole subunit and thiazole subunits [10]. The other bioactive peptides, e.g., Microcin B17 (**8**), show an oxazole linked to thiazole. Moieties like these can be useful pharmacophores in combinatorial libraries since their activity appears to correlate with the location and identity of the tandem pairs [7]. All the amino acids present in **38** show an L-configuration. This compound is cytotoxic towards the HMO2, HepG2, and Huh7 cell lines [10]. No antiviral activity has been reported for **38** [10]; however, its analogue, which contains the 4,2-bisheterocyclic tandem pairs, shows antiviral activity and therefore emerges as a useful scaffold for the synthesis of new antiviral agents [57]. 

### 4.10. YM-216391 

**Figure 16:**
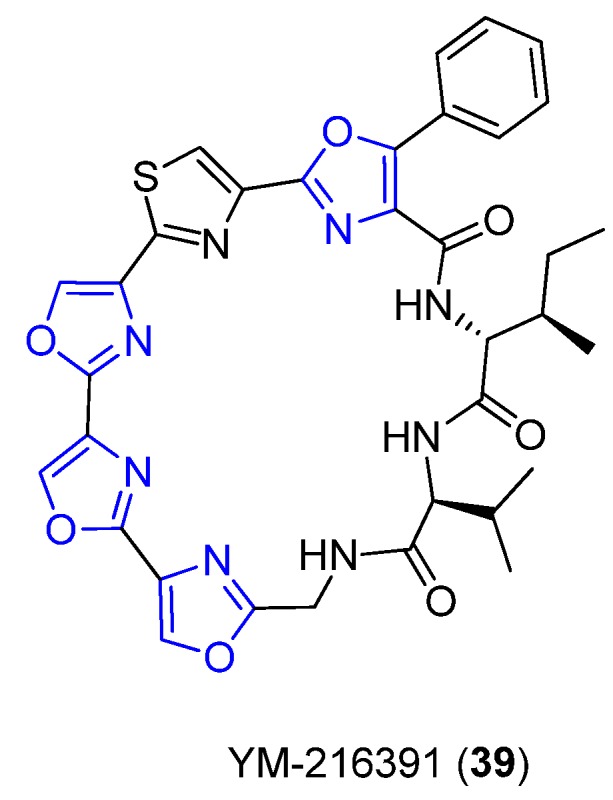
Structure of YM-216391.

YM-216391 (**39**) is an unusual polyoxazole–thiazole-based cyclopeptide that was first isolated from *Streptomyces nobilis* in 2005 by Sohda et al. during the screening of anticancer drugs (Figure 16) [58]. The structure of **39** comprises a sequence of five azoles (trisoxazole, a thiazole, and phenyloxazole), originated from Ser, Cys, and Phe, respectively, linked via a Gly–Val–Ile tripeptide tether [58]. Compound **39** shares both the structure and biological homology with Telomestatin [58] a potent telomerase inhibitor isolated from *Streptomyces anulatus* with promising potential use in cancer chemotherapy. Biological experiments suggest that **39** is cytotoxic towards human cervical cancer HeLa S3 cells and other human cancer cell lines. The OVCAR-3 cell line is more sensitive to **39** than other cell lines, while colon cancer KM-12 and HCT-15 cell lines are more resistant than the other cell lines used in the screening [58]. 

### 4.11. Urukthapelstatin A 

**Figure 17:**
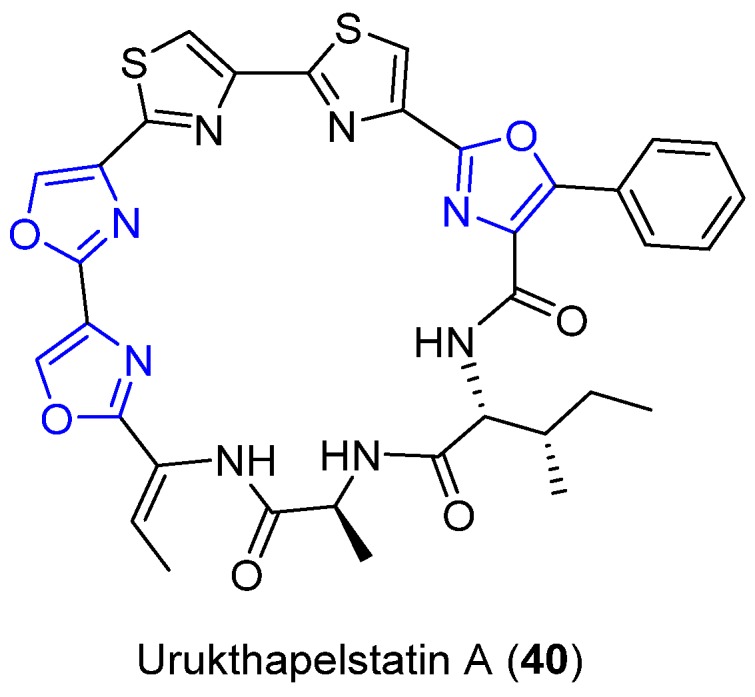
Structure of Urukthapelstatin A.

Urukthapelstatin A (Ustat A) (**40**) is a unique polyoxazole–thiazole-based cyclic peptide that was discovered by Matsuo et al (Figure 17) [59]. It derives from the Thermoactinomycetaceae family and genus *Mechercharimyces asporophorigenens* YM11-542 and was detected during a broad screening of antibiotics. The structure of **40** resembles that of Mechercharmycin **41** and YM-216391 (**39**), potent cytotoxic compounds isolated from the Thermoactinomycetaceae and *Streptomyces*, respectively. Showing strong cytotoxicity at the nanomolar range against human cancer cell lines, especially lung and ovarian cancer cell lines, **40** also displays activity similar to other biologically active drugs currently in use [59]. 

### 4.12. Mechercharmycins A and B

**Figure 18:**
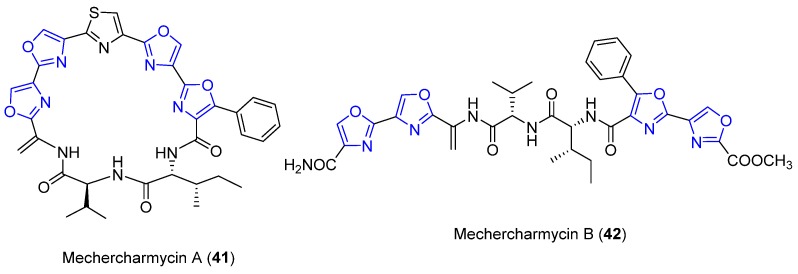
Structures of Mechercharmycin A and B.

Mechercharmycins A (**41**) and B (**42**) were isolated from Thermoactinomyces sp. YM3-251 (Figure 18), collected in Mecherchar in the Republic of Palau (North Pacific Ocean) by Kanoh et al. [60] during the screening of marine microorganisms with antitumor activity.

X-ray crystallographic studies and 2D NMR revealed that **41** is a macrocycle with a peptidic nature composed of dehydroalanine, Val and Ile, and five continuum azoles (oxazoles and thiazole) [60]. Compound **41** has a similar structure to that of IB-01211 [61] isolated from the closely related marine microorganism strain ES7-008. Inactive against cancer cells, **42** is a linear congener of the antitumor active **41**. This observation may imply that the cyclic nature of **41** is essential for its antitumor activity. Furthermore, the structure and biological activity of **41** closely resemble those of the strongly cytotoxic peptides YM-216391 (**39**) and Ustat A (**40**) [60].

### 4.13. Haliclonamides A–E 

**Figure 19:**
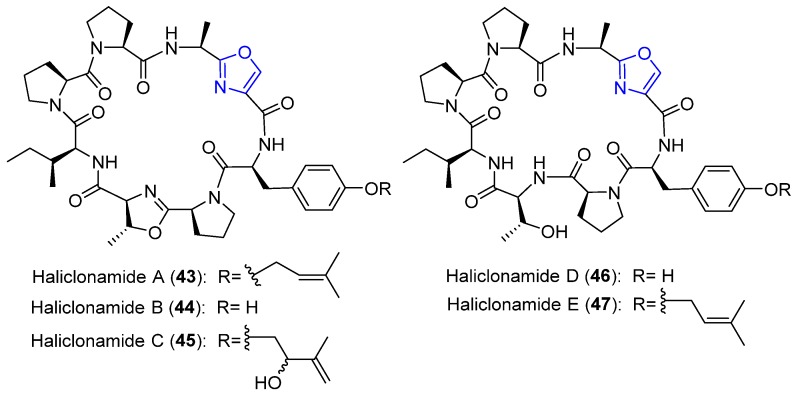
Structures of Haliclonamide A–E.

Marine sponges were studied to determine whether they contained iron-binding natural compounds. This study was conducted to further understand iron-related biofunctions in living organisms in seawater, since the world’s oceans are iron-deficient environments and more than 99% of dissolved iron in ocean water is bound to organic substances [62,63,64,65]. Haliclonamide A (**43**) and its analogue Haliclonamide B (**44**) were isolated from the Palauan marine sponge *Haliclona* sp. by Guan et al. The spectroscopic analysis established that the structures of **43** and **44** contain methlyoxazoline and oxazole moieties. The structure of **43** was determined as a novel cyclic peptide containing oxazole and methyloxazoline rings (Figure 19). 

Peptides **43** and **44** form a 1:1 stable complex specifically with trivalent iron Fe(III) and Cr(III). However, they fail to form complexes with divalent iron and other cation species such as Cu^2+^, Zn^2+^, Co^2+^, Ni^2+^, Al^3+^, or Ti^3+^. The iron concentration in *Haliclona* sponge is from 10 to 100 times more concentrated than in other seawater organisms/tissues. This observation suggests that this sponge takes up iron through non-siderophore metal-binding of **43** and **44**. Other marine organisms may also take up iron in a similar manner [66]. 

Haliclonamides C (**45**), D (**46**), and E (**47**) were isolated from the Palauan marine sponge *Haliclona* sp. by Sera et al. while searching for new bioactive peptides with antifouling activity [67]. These peptides show antifouling activity towards the blue mussel *Mytilus edulis galloprovincialis*. These antifouling activities were tested by the foot-stimulating method developed by Hayashi and Miki in 1996 [68]. The structures of **45**–**47** were determined by spectroscopic analysis (Figure 19) [67]. 

### 4.14. Myriastramides A–C

**Figure 20:**
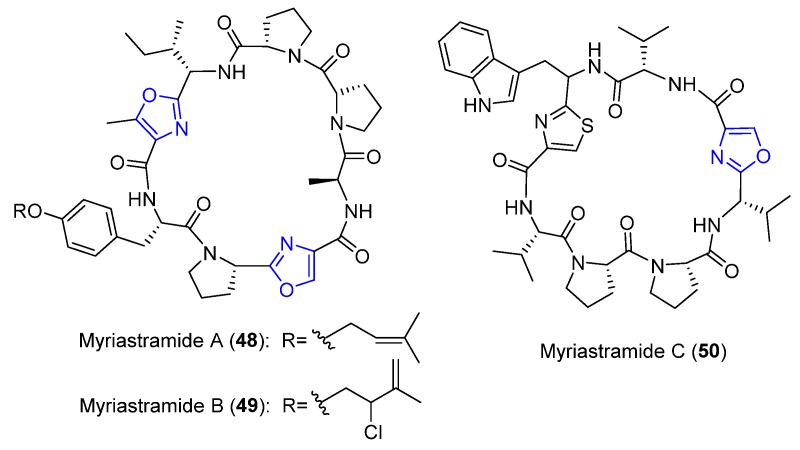
Structures of Myriastramide A–C.

Myriastramides A–C (**48**–**50**) are modified cyclic cynobactin-like octapeptides (Figure 20). They were isolated by Erickson et al. from *Myriastra clavosa* (Order Astrophorida, Family Ancorinidae), collected in the Philippines. Produced via polyketide biosynthesis, **48**–**50** are unexpectedly biologically inactive, in spite of being isolated from cytotoxic fractions of *M. clavosa* extracts. The structures of **48**–**50** were elucidated by NMR spectroscopic analyses, as well as by degradation and derivatization studies. The structure of **48** contains Ile, O-substituted Tyr, Ala, three Pro residues, a prenyl ether moiety, an oxazole, and a methyloxazole ring. Compounds **48** and **49** differ only in the R groups. Myriastramide C (**50**) was found to be unstable, decomposing upon storage. Its structure has a Trp, two Pro residues, and three Val residues, thiazole and oxazole rings [69]. Importantly, the structures of **48** and **49** show a similar composition to that of the haliclonamides. However, the latter differ regarding the amino acid sequence, N-to-C linkage, and the partial reduction of the methyloxazole present [66]. Compound **48** shows L-stereochemistry at the α-position of all the residues, as determined by Marfey’s method. The Ile was analyzed by ligand-exchange, a chiral HPLC method, which confirmed the L-Ile configuration [69].

### 4.15. Wewakazoles 

**Figure 21:**
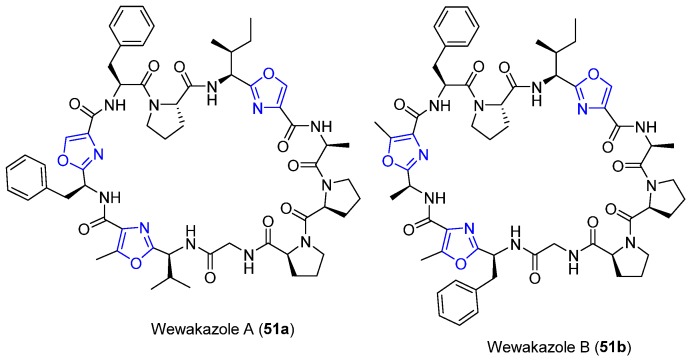
Structures of Wewakazole A and Wewakazole B.

Wewakazole A (**51a**) and Wewakazole B (**51b**) (Figure 21) are cyanobactins that were isolated from Red Sea *Moorea producens* by Lopez et al. and from a Papua New Guinea collection of *Lyngbya majuscula* by Nogle et al., respectively [13,70]. These two cyclic dodecapeptides contain nine common amino acids and three oxazole rings. The difference between **51a** and **51b** is that one has an oxazole moiety instead of a methyl oxazole and a Val rather than a second Ala [13]. **51b** is more cytotoxic towards numerous cancer cell lines than **51a** [13,71,72]. 

### 4.16. Keramamides B–E 

**Figure 22:**
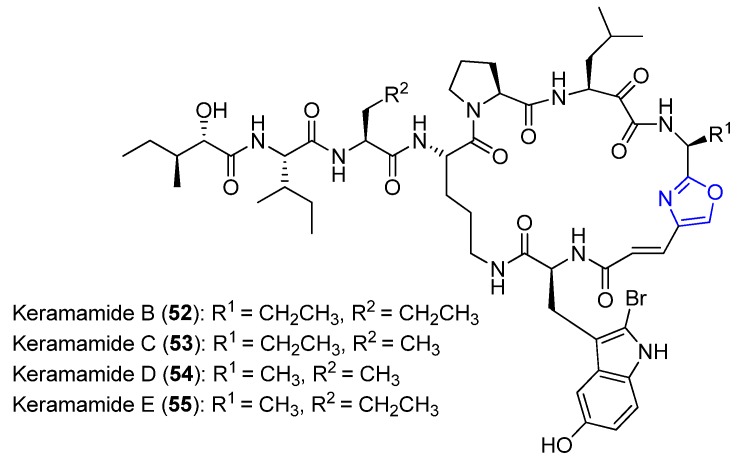
Structures of Keramamide B–E.

The structure of Keramamide B (**52**) [73] has three non-proteinogenic amino acids—ornithine (Orn), α-aminobutyric acid (Aba), and norvaline (nVal) (Figure 22). This compound is a sidechain-to-tail cyclic peptide that features a rare conjugation of three modified amino acid units (α,β-unsaturated amide functionality, an oxazole ring, and an α-keto-β-amino acid moiety) linked consecutively within the cyclic scaffold. Additionally, the units nVal and Orn are important building blocks in the structure of **52**, in that the former is converted into the oxazole ring, while the α-NH of the latter bears the side-chain part of the peptide, and the δ-NH, which is bonded to the carbonyl of the 2-bromo-5-hydroxytryptophan (BhTrp) group, forms part of the macrocyclic peptide backbone. Another uncommon structural characteristic of **52**, apart from the nVal in between the Ile and Orn residues, is the presence of the 2-hydroxy-3-methylpentanoic acid (Hmp) group protecting the N-terminus in the side chain. Moreover, the marine sponge *Theonella* [73,74], from which **52** was obtained, also furnished Keramamides C–E (**53**–**55**), among others lacking the oxazole ring, such as Keramamide A [75]. Although the structures of **52**–**55** all present identical modified amino acid components, a major difference regards the type of R^1^ and R^2^ substituents. Of the first keramamides isolated, **52**–**54,** and tested for the inhibition of superoxides, generated by human neutrophils, all of them displayed biological activity; **55**, in turn, showed antitumor activity on L1210 murine leukemia cells and KB human epidermoid carcinoma cells (Figure 22) [74].

## 5. Bicyclic Peptides

### Diazonamides A–E 

**Figure 23:**
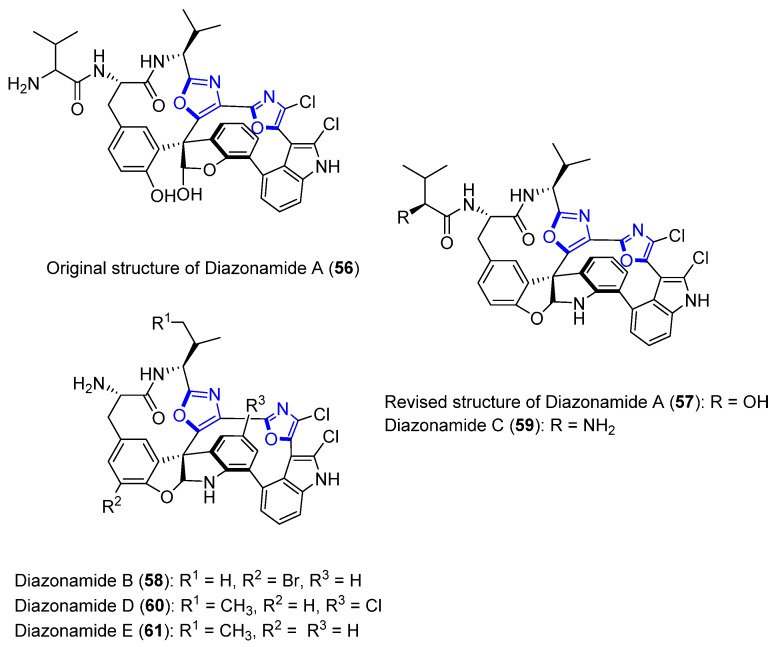
Structures of Diazonamide A–E.

Diazonamides A and B (**56** and **58**) were originally isolated by Lindquist et al. from the colonial ascidian *Diazona chinensis* (revised to *Diazona angulata* [76]) (Figure 23). Spectroscopic characterization revealed that these compounds had complex structures [77]. In particular, the residues Tyr and Val (linked to the oxazole ring) were found to have an L-configuration, but no definitive stereochemical assignment was provided for C–OH in the furan ring or for the terminal Val residue in **56**. However, a synthetic program devoted to **56** reassigned the structure of this compound to that of Diazonamide A (**57**), affording further information on the chemical composition and on the stereochemistry of **57**. Other diazonamides C–E (**59**–**61**) were studied from the ascidian *Diazona angulate* [76]. Compounds **57**–**61** are post-translationally modified peptides, but they also contain three natural amino acids (Val, Trp, and Tyr). The structural complexity and uniqueness of **57**–**61** is highlighted by the presence of chlorinated oxazole as part of the bis-oxazole moiety, the chloroindole unit, and the fragment (*b*)-fused dihydrobenzofuran–dihydroindole, all merged within a strained double macrocyclic array. The metabolites **59**–**61** showed moderate cytotoxicity in three human tumor cell lines [76]. Regarding **57**, it was found to be more cytotoxic than **58** [77]. 

## 6. Thiopeptides

Thiopeptides are naturally occurring peptides of ribosomal origin. They are characterized by a dehydroamino acid tail, with a central nitrogen-containing 6-membered heterocycle, and several azoles in a macrocyclic array. Most thiopeptides show strong inhibition of protein synthesis in bacteria and share a common mode of action [78].

### 6.1. Baringolin

**Figure 24:**
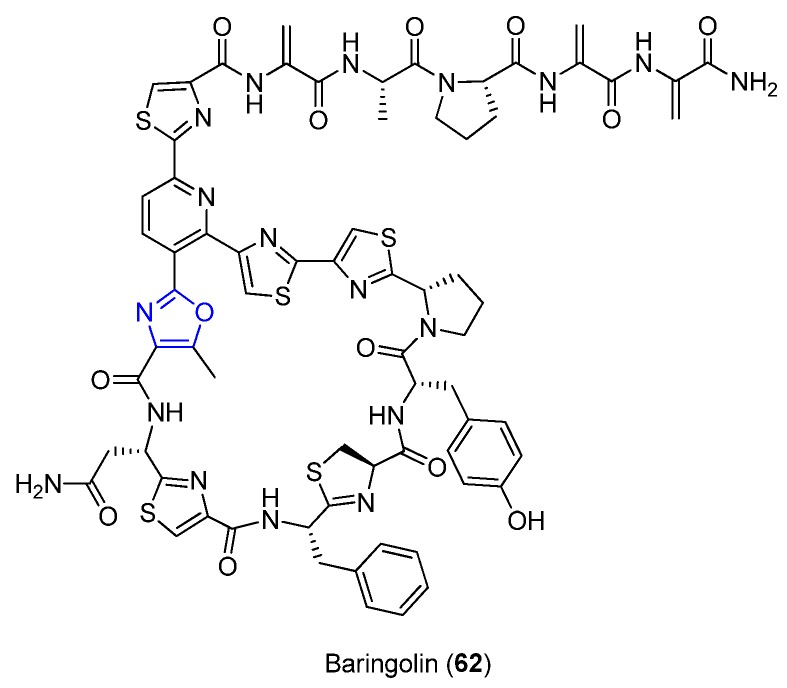
Structure of Baringolin.

Baringolin (**62**) was isolated by a pharmaceutical company, the Instituto Biomar SA (Figure 24), from the marine Micrococcaceae family and bacterium *Kucuria* sp. MI-67-EC3-038 strain, collected in Alicante, southern Spain [79]. Baringolin **62** is a naturally occurring peptide that belongs to the thiopeptide family of *d* series [80], and it contains a central 2,3,6-trisubstituted pyridine [80]. It is considered a member of the thiopeptide family of antibiotics because of the presence of a dehydroalanine side chain, high sulfur content, and antibacterial activity commonly found in thiopeptide [81].

The spectroscopic methods used to elucidate the structure of **62** assigned two parts: the pentapeptide and the macrocycle. The macrocycle has three natural amino acids (Tyr, Phe, and Asn), a pyridine, three thiazole rings, a methyloxazole ring, and other structural motifs not present in other thiopeptides, such as a thiazoline with a chiral center and a pyrrolidine motif derived from a Pro residue. Compound **62** also has a long peptidic tail—a pentapeptide with three methylidenes—attached to the pyridine through the fourth thiazole ring. Additionally, the structure of **62** features the longest tail reported to date in the described family of antibiotics. It also contains seven stereocenters with natural L-configuration. These findings are consistent with the ribosomal origin of **62**. This compound shows good activity against Gram-positive bacteria, including *Propionibacterium acnes*, *Micrococcus luteus*, *S. aureus*, and *B. subtilis* [79].

Other members of the thiopeptide family, discussed below, include the following natural metabolites: Thioxamycin **63**; Thioactin **64**; Promothiocins **65** and **66**; Sulfomycins **67**–**69**; A10225 (**70**–**72**); and Methylsulfomycin **73**. 

### 6.2. Thioxamycin and Thioactin

**Figure 25:**
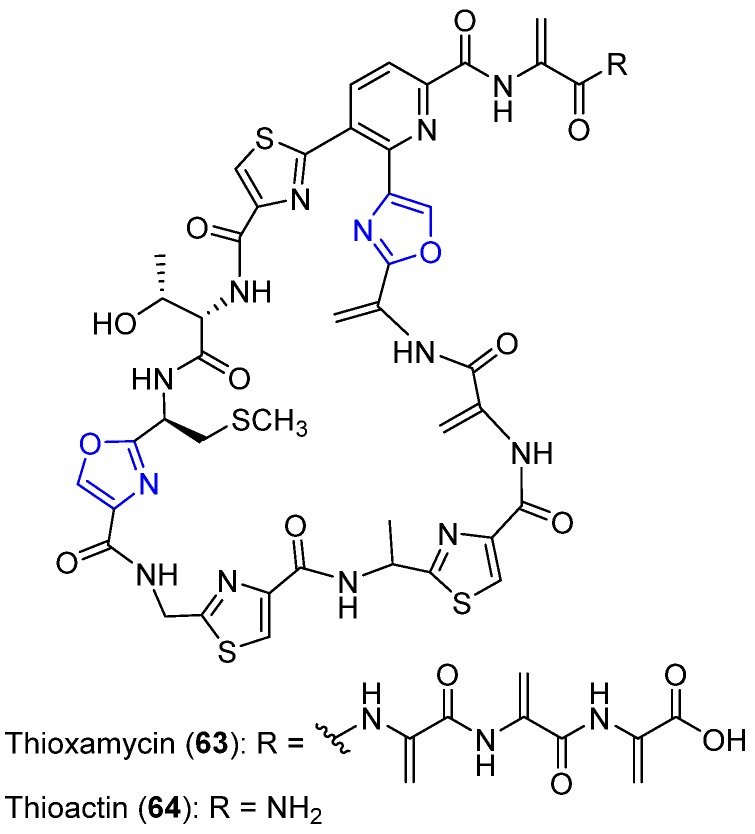
Structures of Thioxamycin and Thioactin.

Matsumoto et al. isolated Thioxamycin **63** (Figure 25) as an acidic, lipophilic sulfur-containing peptide from the *Streptomyces* sp. strain PA-46025. No stereochemical information was provided for the thiazole-modified Ala residue [82]. Compound **63** exhibits a wide range of antibacterial activity against anaerobic Gram-positive and Gram-negative bacteria, as well as against aerobic Gram-positive bacteria. With a high sulfur content, **63** is closely related to Sulfomycins **67** and **68** (see below), which show antibiotic properties [82]. Thioactin **64** was also isolated as a derivative of **63** by Yun et al. from *Streptomyces* sp. strain DP94. Using 1D and 2D NMR spectra, those authors revealed that the structure of **64** contains Thr, two oxazole rings, three thiazole rings, and two dehydroalanines (Figure 25). 

Compounds **63** and **64** share similar structural features. However, the C-terminal carboxylic acid of the dehydroalanine side chain in **63** is replaced with an amide group in **64** [83]. 

### 6.3. Promothiocins A and B 

**Figure 26:**
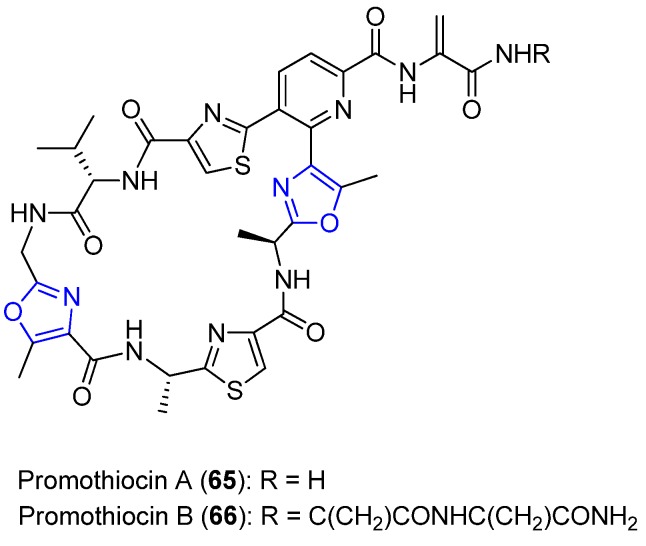
Structures of Promothiocin A and B.

Promothiocins A (**65**) and B (**66**) were isolated from *Streptomyces* sp. SF2741 by Yun et al. as tipA promoter-inducing substances (Figure 26) [81]. TipA is a regulatory protein that autonomously activates the transcription of its promoter when interacting either with thiostrepton [84,85] or other related thiopeptide antibiotics [86]. By means of 1D and 2D NMR spectra, the planar structures of **65** and **66** were assigned as unique 26-membered thiopeptides composed of Val, Gly, Ala, two methyloxazole rings, two thiazole rings, pyridine, and three dehydroalanines [81]. The absolute stereochemistry of **65** and **66** has been examined in degradation studies. The residues Ala and Val were found to have an L-configuration [87]. 

### 6.4. Sulfomycins I–III 

**Figure 27:**
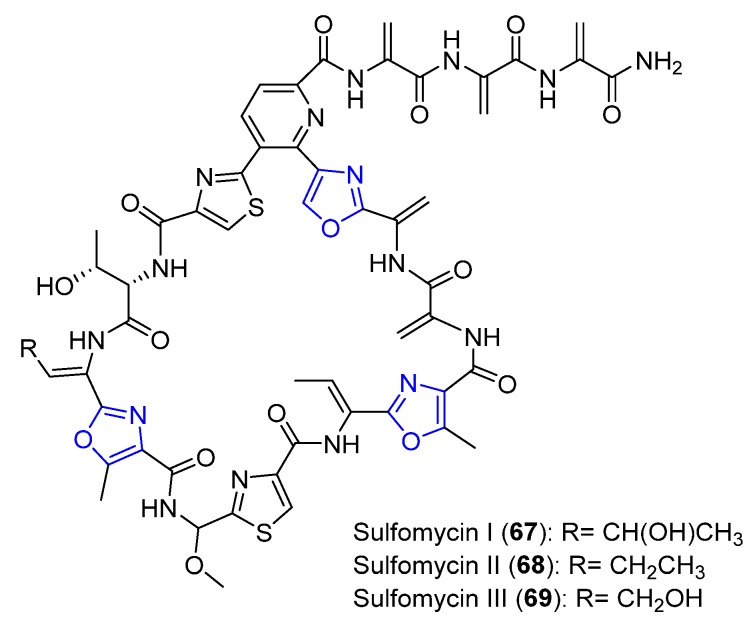
Sctructures of Sulfomycins I, II, and III.

Sulfomycins I–III (**67**–**69**) were first isolated by Egawa et al. from *Streptomyces viridochromogenes* MCRL-0368 (Figure 27) [88]. These compounds exhibited antibacterial activity against Gram-positive cocci, bacilli, mycoplasma, and anaerobic bacteria, as well as against those resistant to penicillin, streptomycin, tetracycline, chloramphenicol, and staphylococci freshly isolated from clinical material [88,89]. They are weakly active against *Neisseria, Bordetella*, and mycobacteria. The structures of **67**–**69** were determined by 1D and 2D NMR spectra. They all contain two thiazole rings, three oxazole rings, four dehydroalanines, Thr and pyridine, and differ only in one of the oxazole units [89,90]. In the structure of **68**, the Thr unit has an L-configuration [89]. Regarding the carbon atom attached to the methoxy group, no stereochemical information was provided.

### 6.5. A10255B, E, and G

**Figure 28:**
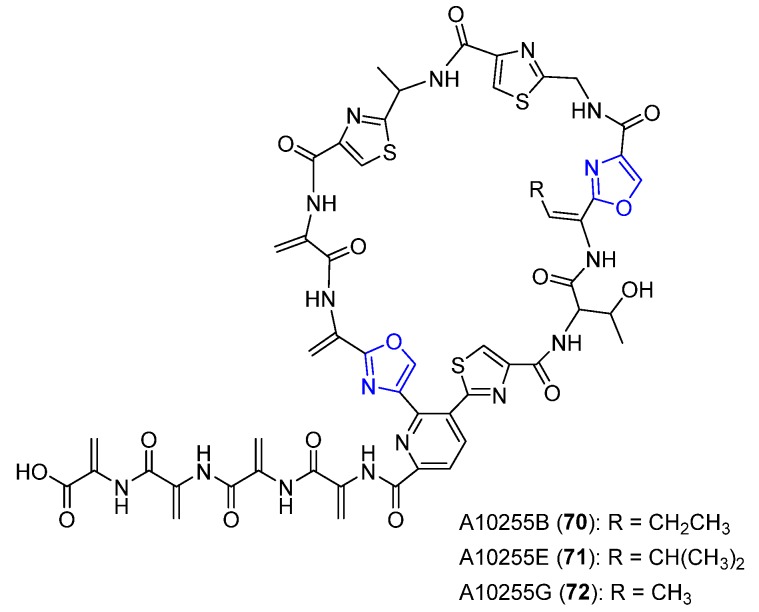
Structures of A10255B, E, and G.

A10255 is a multicomponent (designated A10255B (**70**), C, E (**71**), F, G (**72**), H, and J) (Figure 28), sulfur-containing complex of antibiotics that was isolated from *Streptomyces gardneri* NRRL 15,537 by Debono et al. These compounds display strong antimicrobial activity against Gram-positive bacteria and have potential use as growth promoters, and they also prevent lactic acidosis in farm animals. The major components of these antibiotics are A10255B (**70**) and G (**72**), which were isolated in sufficient amount to allow the determination of their structures, while the other components were isolated in small amounts. The structures of compounds **70**–**72** are similar to the highly modified thiopeptide family of antibiotics, e.g., the metabolites Sulfomycin **67**, Thioxamycin **63**, Berninamycin A [90], Thiopeptin [91], and Thiostrepton [84,85]. A10255 (**70**–**72**) share common features with these thiopeptides, such as oxazole/thiazole rings, a pyridine moiety, and several unusual amino acid units, such as dehydrovaline, dehydrobutyrine, and dehydroalanine [92]. 

Compounds **70**–**72** show a similar composition, except for the alkyl substituents at R located in one of the oxazole-containing moieties. Compound **72** contains a masked dehydrobutyrine residue (R-methyl group), **70** has a masked dehydronorvaline residue (R-ethyl group), while **71** contains a masked dehydronorleucine residue (R-isopropyl group) [93]. No stereochemical assignment of the Thr and the masked Ala was provided by the authors.

### 6.6. Methylsulfomycin I

**Figure 29:**
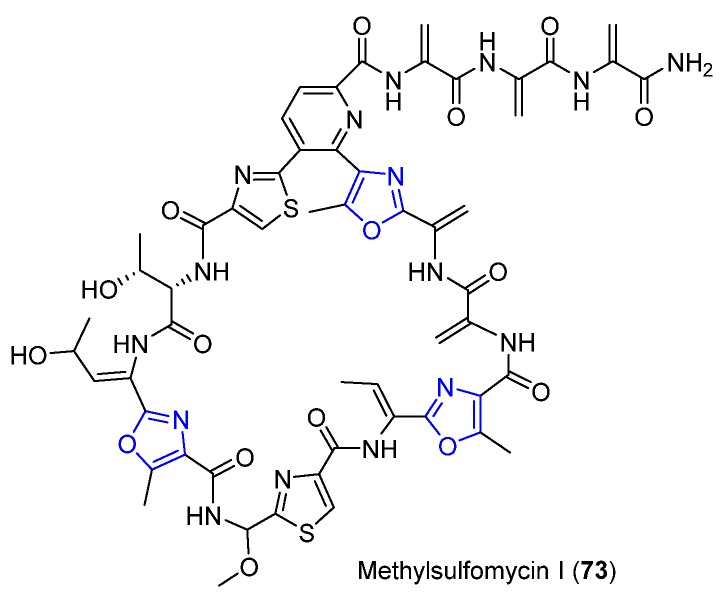
Structure of Methylsulfomycin I.

Methylsulfomycin I (**73**) is a new cyclic peptide antibiotic that was isolated from the fermentation broth of *Streptomyces* sp. HIL Y-9420704 by Kumar et al. (Figure 29). Compound **73** shows good in vitro activity for a wide range of Gram-positive bacteria, as well as for vancomycin- and teicoplanin-resistant organisms. It was found to be 800-fold more effective than vancomycin (>100 µg/mL) when tested against vancomycin- and teicoplanin-sensitive and -resistant organisms. Compound **73** decomposes into a practically insoluble biologically inactive compound in most organic solvents commonly used. The structure of **73** was elucidated by NMR and GC-MS. Like other thiopeptides, it contains a macrocyclic core and a dehydro tail, in addition to two thiazole rings, three oxazole rings, a Thr residue with other unidentified units, a pyridine, and four dehydroalanines. The NMR spectral data of this compound are closely related to those reported for other thiopeptides, such as Thioxamycin **63**, Berninamycin A [90], and Sulfomycin (I) **67**. Compound **73** is similar to Sulfomycin **67** except that it has three 5-methyloxazole rings [94]. 

## 7. Overview

This work provides general information on the isolation, structural elucidation, and possible biological activity shown by natural peptides containing oxazole moieties and highlights the importance of oxazole rings in peptides (see Table 1 for a list of the peptides discussed in this work).

The marine environment is home to various species, such as algae, sponges, and cyanobacteria, which naturally synthesize unique bioactive oxazole-containing peptides. Overall, these oxazoles range from short linear peptides to long and complex structures. Other organisms produce cyclic peptides, bicyclic peptides, and even the so-called thiopeptides. Oxazoles and thiazoles form part of the macrocyclic structures of these three classes of peptides, which also contain a pyridine moiety in the case of thiopeptides. 

Finally, this work reaffirms the marine environment as a rich source of new compounds with biological properties of interest—compounds expected to find application in the design of novel drugs in the near future.

## Figures and Tables

**Table 1:** Brief information about the source, isolation, bioactivity, and references of oxazole-containing peptides.

Peptide Name	Natural Source	Organism	Bioactivity	Class/Type	References
A10225B, G and E	Colorado soil bacterium	*Streptomyces gardneri* NRRL 15537	Antimicrobial activity, growth promoter, prevent lactic acidosis in farm animals	Thiopeptides	[92,93]
Aerucyclamides C	Cyanobacterium	*Microcystis aeruginosa* PCC 7806	Promising antimalarial agent	Cyclic peptide	[56]
Almazoles A–D	Senegal seaweed	Genus *Haraldiophyllum*	Almazole D: antibacterial activity	Short linear peptide	[18,19,20,21,22]
Baringolin	Spain bacterium	*Kucuria* sp. MI-67-EC3-038	Antibacterial activity	Thiopeptide	[78,79]
Bistratamides	Philippine ascidian	*Lissoclinum bistratum*	Bistratamide D: neurodepressant properties Bistratamides M and N: with antitumor activity	Cyclic peptides	[39,40,41]
Dendroamides A–C	Terrestrial blue-green alga	*Stigonema dendroideum*	Dendroamide A: potent MDR-reversing agent	Cyclic peptide	[43,44,45]
Diazonamides A–E	Marine colonial ascidian	*Diazona angulata*	Cytotoxicity activity	Bicyclic peptide	[76,77]
Dolastatins E and I	Japanese sea hare	*Dolabella auricularia*	Cytotoxicity activity	Cyclic hexapeptides	[49,51]
Goadsporin	Soil bacterium	*Streptomyces* sp. TP-A0584	Antibacterial activity	Long linear peptide	[37,38]
Haliclonamides A–E	Palauan marine sponge	*Haliclona* sp.	Haliclona A and B: Metal-Iron uptake Haliclonamide C–E: antifouling activity	Cyclic peptides	[62,63,64,65,66,67]
Keramamides	Okinawan marine sponge	*Theonella* sp.	Keramamide B–D: inhibits superoxides Keramamide E: antitumor activity	Cyclic peptides	[73,74,75]
Leucamide A	Australian marine sponge	*Leucetta microraphis*	Cytotoxicity activity	Cyclic peptide	[10,57]
Martefragin A	Marine Red algae	*Martensia fragilis*	Inhibit lipid peroxidation	Short linear peptides	[23,24]
Mechercharmycins A and B	Marine bacterium	*Thermoactinomyces* sp. YM3-251	Mechercharmycins A: antitumor activity	Cyclic peptides	[60]
Methylsulfomycin I	Soil bacterium	*Streptomyces* sp. HIL Y-9420704	Antibacterial activity	Thiopeptide	[94]
Microcyclamides 7806 A and B	Cyanobacterium	*Microcystis aeruginosa* NIES-298 and PCC-7806	Microcyclamide: Cytotoxicity activity	Cyclic peptides	[54,55]
Microcin B17	Soil bacterium	*Escherichia coli*	Poison DNA gyrase	Long linear peptide	[29,30,31,32,33,34]
Muscoride A	Cyanobacterium	*Nostoc muscorum*	Antibacterial activity	Short linear peptide	[25,26,27,28]
Myriastramides	Cyanobacterium	*Myriastra clavosa*	No biological activity mentioned on literature	Cyclic peptides	[69]
Nostocyclamide, Nostocyclamide M	Cyanobacterium	*Nostoc* 31	Both have cyanobacterial and allelopathic activity Nostocyclamide M: anti-algal activity	Cyclic peptides	[12,47]
Plantazolicins A and B	Soil bacterium	*Bacillus amyloliquifaciens* FZB42	Plantazolicin A: Antibacterial activity	Long linear peptides	[34,35,36]
Promothiocins A and B	Soil bacterium	*Streptomyces* sp. SF2741	Not reported in literature	Thiopeptide	[81,87]
Raocyclamides A and B	Cyanobacterium	*Oscillatoria raoi*	Raocyclamide A: Cytotoxicity activity	Cyclic peptide	[52,53]
Sulfomycins I-III	Soil bacterium	*Streptomyces viridochromogenes* MCRL-0368	Antibacterial activity	Thiopeptide	[88,89,90]
Tenuecyclamides A–D	Cyanobacterium	*Nostoc spongiaeforme* var. *tenue* Rao	Antimicrobial and cytotoxicity activity	Cyclic peptides	[48]
Thioxamycin and Thioactin	Soil bacterium	*Streptomyces* sp. strain PA-46025	Antibacterial activity	Thiopeptide	[82,83]
Urukthapelstatin A	Marine bacterium	*Mechercharimyces asporophorigenens* YM11-542	Cytotoxicity activity	Cyclic peptide	[59]
Venturamides A and B	Cyanobacterium	*Oscillatoria* sp.	Antimalarial activity	Cyclic peptides	[11]
Wewakazole A and B	Cyanobacterium	*Lyngbya majuscula, Moorea producens*	Cytotoxicity activity	Cyclic peptides	[13,71,72]
YM-216391	Soil bacterium	*Streptomyces nobilis*	Cytotoxicity activity	Cyclic peptide	[58]
